# Sorafenib and docosahexaenoic acid act in synergy to suppress cancer cell viability: a role of heme oxygenase 1

**DOI:** 10.1186/s12885-018-4946-9

**Published:** 2018-10-26

**Authors:** Yang Jiao, Tanya Watts, Jing Xue, Bethany Hannafon, Wei-Qun Ding

**Affiliations:** 1 Department of Pathology, University of Oklahoma Health Sciences Center, 975 NE 10th Street, BRC 411A, Oklahoma City, OK 73104 USA; 2 Department of Radiation Genetics, School of Radiation Medicine and Protection, Medical College of Soochow University, Suzhou, China; 3 Suzhou Cancer Center Core Laboratory, Nanjing Medical University Affiliated Suzhou Hospital, Suzhou, Jiangsu China; 4 Peggy and Charles Stephenson Cancer Center, Oklahoma City, 73104 USA

**Keywords:** Sorafenib, Docosahexaenoic acid, Cancer, Heme oxygenase 1, Synergy

## Abstract

**Background:**

Docosahexaenoic acid (DHA) is a long chain n-3 polyunsaturated fatty acid that has anticancer activity. Heme oxygenase 1 (HO-1) is a potential therapeutic target due to its cytoprotective activity in cancer cells. We recently reported that DHA induces HO-1 gene transcription in human cancer cells by augmenting the degradation of Bach1 protein, which functions as a negative regulator of HO-1. Since the degradation of Bach1 protein relies on protein phosphorylation, we hypothesized that DHA-induced HO-1 gene transcription could be attenuated by kinase inhibitors, resulting in an enhanced cytotoxicity. Sorafenib, a tyrosine kinase inhibitor, was first applied to test our hypothesis.

**Methods:**

Human cancer cell lines and a xenograft nude mouse model were applied to test our hypothesis. Gene expression was analyzed by western blot analysis and reporter gene assay. Cell viability was analyzed using a colorimetric assay. Isobologram was applied to analyze drug action.

**Results:**

Pretreatment of cancer cells with Sorafenib significantly attenuated DHA-induced degradation of Bach1 protein. Consequently, DHA-induced HO-1 gene transcription was reversed by Sorafenib as evidenced by western blot and reporter gene analysis. Sorafenib acted synergistically with DHA to suppress cancer cell viability in various human cancer cell lines and suppressed tumor xenograft growth in mice fed a fish oil enriched diet (high n-3/DHA), as compared to mice fed a corn oil (high n-6) diet. Screening of the NCI-Oncology Drug Set IV identified a group of anticancer compounds, including Sorafenib, which enhanced DHA’s cytotoxicity, as well as a set of compounds that attenuated DHA’s cytotoxicity.

**Conclusions:**

We demonstrate that sorafenib attenuates DHA-induced HO-1 expression and acts in synergy with DHA to suppress cancer cell viability and tumor growth. Considering the known health benefits of DHA and the clinical effectiveness of Sorafenib, their combination is an attractive therapeutic strategy against cancer.

## Background

Docosahexaenoic acid (DHA), a long chain n-3 polyunsaturated fatty acid, has anticancer activity in various experimental model systems [1–6]. Dietary intake of DHA also has many health benefits to humans, such as lowing blood lipid levels, preventing cardiovascular disorders [7], and nurturing the central nervous system [8]. DHA is currently prescribed for the treatment of lipid and cardiovascular disease [9]. The unique features of DHA, having both anticancer activity and health benefits to humans, indicates a potential strategy against cancer by combining DHA and other cancer therapeutics [10]. In this context, DHA has been shown to enhance the anticancer activity of various chemotherapeutic drugs [11], and is currently being tested in clinical trials for combination therapy [12]. However, while the idea of combining DHA and other anticancer drugs for cancer therapy is well conceived, the mechanism of how DHA may augment the anticancer action of cancer therapeutics remains elusive.

We have previously reported that DHA’s anticancer activity can be, in part, explained by enhancement of oxidative stress in cancer cells [13]. These observations are supported by other reports using different cancer model systems [10, 14–16]. We have demonstrated that the enzymatic antioxidant response system in cancer cells plays an important role in mediating DHA’s anticancer action. Specifically, when the antioxidant response system is activated in cancer cells, the cytotoxicity of DHA is attenuated, whereas the opposite is true when the antioxidant response system is inactivated [13, 17]. One of the established antioxidant response enzymes is heme oxygenase 1 (HO-1) [18]. HO-1 expression is tightly controlled by the Nrf2 signaling pathway [19] and is involved in resistance to chemotherapy [20]. Therefore, targeting HO-1 is a potential therapeutic strategy against cancer. [21, 22]. In our most recent report, we demonstrated that DHA induces HO-1 gene transcription largely by promoting nuclear exportation and degradation of the Bach1 protein [23], a transcriptional repressor that competes with Nrf2 for binding to the Antioxidant Response Elements present in the HO-1 gene promoter [24, 25]. Bach1 nuclear exportation and subsequent degradation occurs after tyrosine phosphorylation of the protein [26]. Therefore, we hypothesized that by blocking Bach1 protein degradation through tyrosine kinase inhibition we could reverse DHA-induced HO-1 expression and more effectively suppress cancer cell viability.

Sorafenib is an established tyrosine kinase inhibitor currently in use or under clinical trial for the treatment of various human cancers [27, 28]. It is therefore an optimal candidate to be applied to test our hypothesis. Our experimental results demonstrate that pretreatment of cancer cells with Sorafenib reverses DHA-induced suppression of nuclear Bach1 expression and attenuate DHA-induced HO-1 gene transcription, resulting in a synergistic action that suppresses cancer cell viability and tumor growth.

## Methods

### Materials

The pGL3/4.5-HO-1 luciferase reporter construct was described in our previous report [23]. The antibodies for Bach1 (sc-14,700) was obtained from Santa Cruz Biotechnology, Inc. (Santa Cruz, CA), and the HO-1 antibody (SPA-896) from Stressgen (Ann Arbor, MI). The Dual-Luciferase Reporter kit was from Promega ([23], Madison, WI). Sorafenib was purchased from LC Laboratories (Woburn, MA). The NCI-Oncology Drug Set IV (101 anticancer compounds) was kindly provided by the Drug Synthesis and Chemistry Branch, Developmental Therapeutics Program, Division of Cancer Treatment and Diagnosis, National Cancer Institute. The β-actin antibody (A5441), DHA, and other chemical agents were analytic grade and purchased from Sigma-Aldrich (St. Louis, MO).

### Cell culture

Human breast cancer cell lines MDA-MB-231 (ATCC® HTB-26™) and MCF7 (ATCC® HTB-22™), and prostate cancer cell line DU-145 (ATCC® HTB-81™) were obtained from the American Type Culture Collection (Manassas, VA, USA). The human ovarian carcinoma cell line A2780 [29] was kindly provided by Dr. Stephen Howell (University of California, San Diego, CA). A2780, MCF7 and MDA-MB-231 cells were cultured in DMEM medium, and DU-145 in EMEM medium, supplemented with 10% fetal bovine serum, 100 IU/ml penicillin, and 100 μg/ml streptomycin. Cells were grown at 37 °C, 5% CO_2_. DHA was prepared and applied as we reported [23]. Sorafenib was dissolved in DMSO at 100 mM. Control cells were treated with vehicle-only. Cell viability was analyzed at the indicated times using the MTS assay (Promega, Madison, WI), as we previously described [30–33].

### Xenograft nude mouse study

Athymic nude mice (Foxn1nu) were purchased from Envigo (United Kingdom) and were used for in vivo evaluation of Sorafenib in accordance with the Institute Animal Care and Use Committee procedures and guidelines. The mice were fed either 7.5% (wt/wt) corn oil diet or 7.5% (wt/wt) fish oil diet (enriched in DHA and eicosapentaenoic acid (EPA) as we recently described [32]) and reported by others [6, 34–38]. A total of 3 × 10^6^ MDA-MB-231 cells were suspended in 100 μL PBS containing 20% Matrigel, and injected s.c. into the flanks of 5-week old female mice. The vehicle (PBS/Cremophor/DMSO = 7.5/2/0.5) and Sorafenib (15 mg/kg) were separately delivered intraperitoneally every two days. Animal body weight and tumor volume were measured three times per week [31]. The tumor volume was calculated using the following formula: *v* = *l* × *w*^*2*^ × 0.5, as we reported [32].

### Western blot analysis

Western blot was performed as described [31]. Cells were lysed, sonicated on ice, and insoluble material was removed by centrifugation. Nuclear protein was extracted, by adding wash buffer containing100 μM PMSF, 200 ng/ml Aprotinin, 1 μg/ml Leupeptin, 200 ng/ml Pepstatin A, and 0.01% NP-40. The lysate was centrifuged and pellets were suspended in buffer containing 25% glycerol, 420 mM NaCl, 1.5 mM MgCl_2_, 0.2 mM EDTA, 0.5 mM DTT, 1 mM PMSF, 2 μg/ml aprotinin, 10 μg/ml leupeptin, 2 μg/ml pepstatin A. The samples were incubated on ice for 30 min and centrifuged to remove insoluble material. Around 40 μg of protein per sample was loaded onto a 10% SDS-PAGE gel, transferred, and blotted with antibodies against HO-1, Bach1, GAPDH, or β-actin.

### Luciferase reporter gene assay

A2780 cells were transfected with the pGL3/4.5-HO-1 luciferase reporter construct using the Fugene HD transfection reagent (Roche, Mannheim, Germany) as previously described [39]. Twenty four hours later, cells were plated into 24-well plates at a density of 2 × 10^5^ per well. Cells were then treated with DHA and/or Sorafenib at the indicated concentration for 18 h. Cell lysates were prepared and luciferase activity assayed [39].

### siRNA knockdown of Bach1

siRNAs targeting Bach1 were purchased from Santa Cruz Biotechnology (Santa Cruz, CA). A2780 cells were co-transfected with 225 nM (final concentration) of Bach1 siRNA, or scrambled non-specific siRNAs (as control) using the Fugene HD transfection reagent (Roche, Mannheim, Germany). The next day, cells were lifted, plated into 96-well plates, and treated with DHA and/or Sorafenib for 48 h. Cell viability was evaluated using the MTS assay. The knockdown of Bach1 was confirmed by Western blot analysis.

### Screening of the oncology drug set IV

The human breast cancer MDA-MB-231 cell line was utilized for drug screening. Cells were plated into a 96-well plate at 8000 per well. Twenty-four hours after plating, 25, 50 or 100 μM DHA was added to cells with or without an oncology compound at the concentration of their IC_25_ or IC_50_ Values. The IC_25_ and IC_50_ values for each compound against MDA-MB-231 cells were obtained by screening the NCI-60 cancer cell panel by the Developmental Therapeutics Program, National Cancer Institute. Forty-eight hours after drug addition, cell viability was analyzed using the MTS assay [30–33]. Compounds were screened in three independent experiments conducted in triplicate, and Clioquinol (10 μM) was applied as a positive control that acts with DHA in synergy to kill cancer cells [30]. Based on the cell viability analysis, compounds were categorized by their ability to enhance, antagonize, or have no effect when combined with DHA. The initial screening tested 101 compounds in the Oncology Drug Set IV, and the results of the initial screen were validated using 45 compounds (limited by availability of the compounds after initial screening).

### Statistical analysis

Statistical analysis was performed with Graphpad Prism software (San Diego, CA). Two-way ANOVA with Bonferroni post-test was used to determine differences among control and experimental groups for combinational drug screening. One-way ANOVA was applied to determine differences among experimental groups for experiments other than drug screening. Isobologram was constructed as we previously described [30].

## Results

### Sorafenib attenuates DHA-induced suppression of Bach1 expression and HO-1 gene transcription

**Fig. 1:**
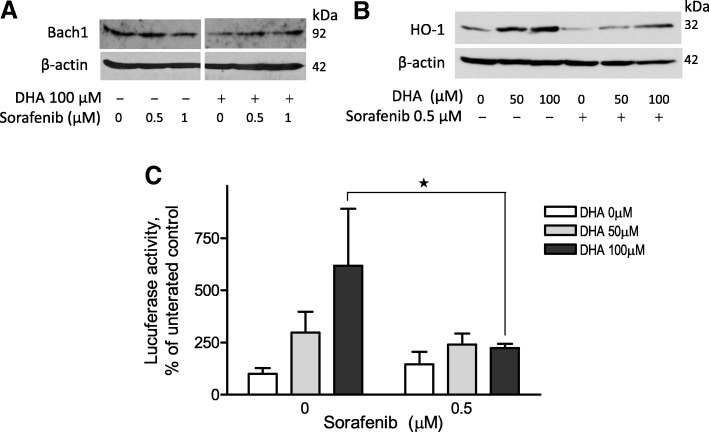
Sorafenib reverses DHA-induced suppression of nuclear Bach1 protein expression and induction of HO-1 transcription in A2780 cells. **a** Cells were pretreated with Sorafenib for 1 h prior to addition of DHA for 18 h. Nuclear proteins were isolated and Bach1 expression was determined by Western blot (*n* = 3). **b** Cells were pretreated with Sorafenib for 1 h prior to addition of DHA for 24 h. Cell lysates were prepared and HO-1 expression was determined by Western blot (*n* = 3). **c** Cells were transfected with the pGL3/4.5-HO-1 luciferase reporter construct and pretreated with Sorafenib for 1 h prior to addition of DHA for 24 h. Luciferase activity was determined and expressed relative to untreated controls (*n* = 3, mean ± SEM, *p < 0.01)

We have previously reported that DHA induces HO-1 gene transcription via Bach1 protein nuclear exportation and degradation [23]. Because Bach1 degradation occurs after tyrosine phosphorylation [26], we tested the hypothesis that the tyrosine kinase inhibitor, Sorafenib, will reverse DHA-induced Bach1 protein degradation and thereby attenuate DHA-induced HO-1 gene transcription. A2780 cells were treated with DHA and Sorafenib at the indicated concentrations for 24 h (Fig. 1). Cellular and nuclear lysates were prepared and analyzed by western blot analysis and reporter gene assay. As shown in Fig. 1a, treatment with 100 μM DHA for 24 h significantly suppressed nuclear Bach1 protein expression in A2780 cells, an observation consistent with our recent report [23]. Pretreatment of A2780 cells with Sorafenib at 0.5–1 μM for 1 h attenuated DHA-induced suppression of Bach1 protein expression, supportive of our hypothesis. Consequently, both basal and DHA-induced HO-1 gene transcription was attenuated by Sorafenib pretreatment, as evidenced by western blot analysis (Fig. 1b). This attenuation was significant when 100 μM DHA was applied in a reporter gene assay (Fig. 1c).

### Sorafenib enhances DHA’s cytotoxicity in human cancer cells

**Fig. 2:**
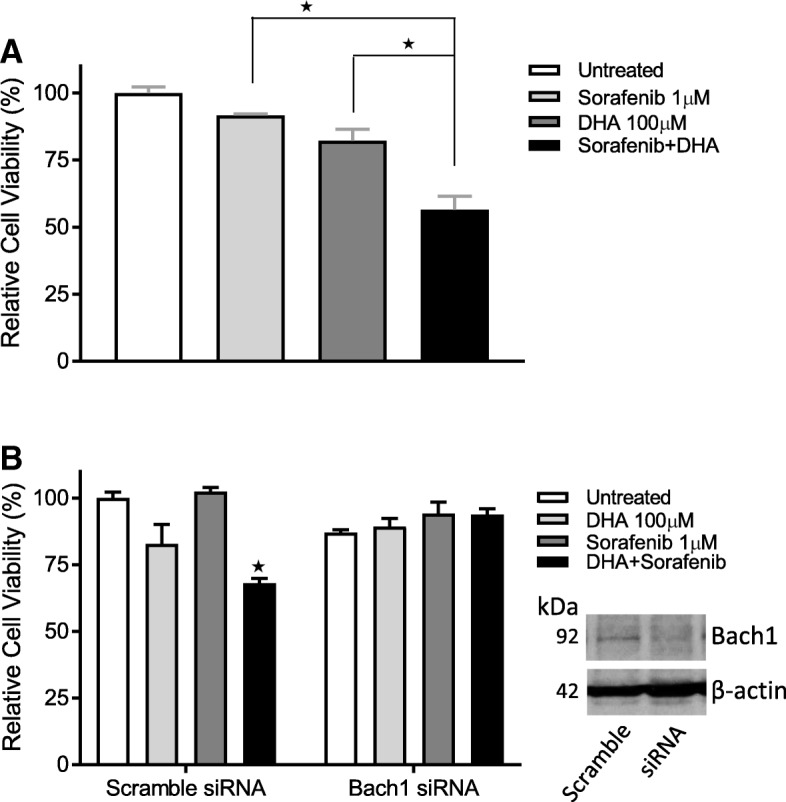
Sorafenib enhances DHA’s cytotoxicity in A2780 cells. **a** Cells were treated with Sorafenib and DHA for 48 h and cell viability was determined by the MTS assay (*n* = 3, mean ± SEM, *p < 0.01). **b** Bach1 was knocked down in A2780 cells using targeted siRNAs. Cells were treated with Sorafenib and DHA for 48 h and cell viability was determined by the MTS assay (*n* = 3, mean ± SEM, *p < 0.01)

**Fig. 3:**
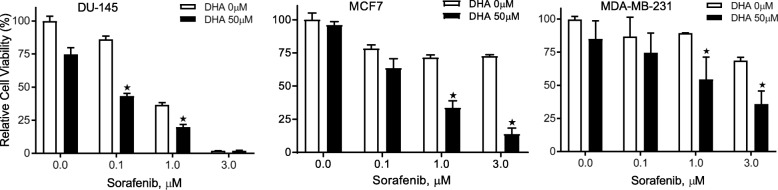
Sorafenib enhances DHA’s cytotoxicity in DU-145, MCF7, and MDA-MB-231 cancer cells. Cells were treated with Sorafenib and/or DHA for 48 h and cell viability was determined by the MTS assay (*n* = 3, means ± SEM, *p < 0.01)

Since HO-1 contributes to chemo resistance in cancer cells [20] and is a potential cancer therapeutic target [21, 22], the attenuation of DHA-induced HO-1 expression levels by Sorafenib could lead to an enhanced cytotoxicity toward cancer cells. This assumption was first confirmed in A2780 cells by MTS assay (Fig. 2a). Treatment with the combination of Sorafenib and DHA for 48 h significantly enhanced their cytotoxicity as compared to that of each compound treatment alone. More importantly, when Bach1 was knocked down in A2780 cells, as shown in Fig. 2b, the cytotoxicity induced by the combination was reversed, suggesting that Bach1 mediates this event. Note that DHA at 100 μM did not cause significant cytotoxicity in A2780 Cells, an observation consistent with our previous reports [13, 17]. To understand whether Sorafenib can also enhance DHA’s cytotoxicity in other human cancer cells, we tested the effect of Sorafenib combined with DHA on cell viability in DU145 (prostate cancer), MCF7 (breast cancer), and MDA-MB-231 (breast cancer) cells. As shown in Fig. 3, the cytotoxicity of DHA and Sorafenib, when used alone, differed among the cell lines, and the combination treatment for 48 h was more cytotoxic than each compound alone in all three cell lines tested, indicating that this effect is not limited to individual cell lines.

### Sorafenib and DHA act in synergy to induce cytotoxicity in MDA-MB-231 cells

**Fig. 4:**
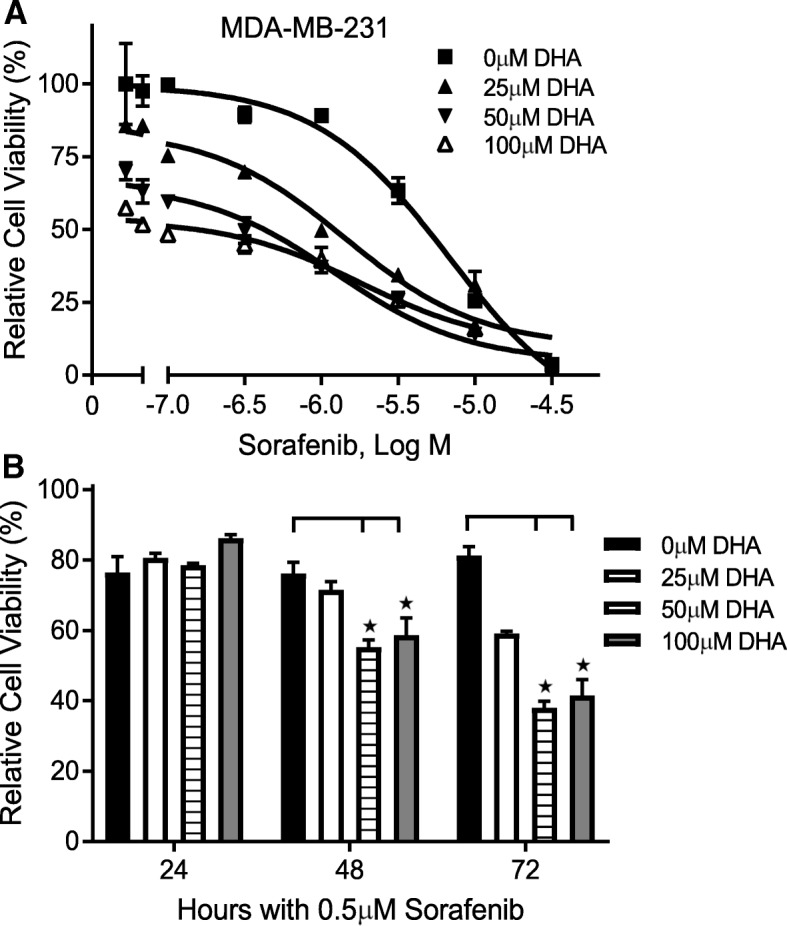
Sorafenib enhances DHA’s cytotoxicity in a concentration- and time-dependent manner in MDA-MB-231 cells. Cells were treated with Sorafenib and DHA at different concentrations for 48 h **a** or for different durations **b** Cell viability was determined by the MTS assay (*n* = 3, means ± SEM, *p < 0.01)

**Fig. 5:**
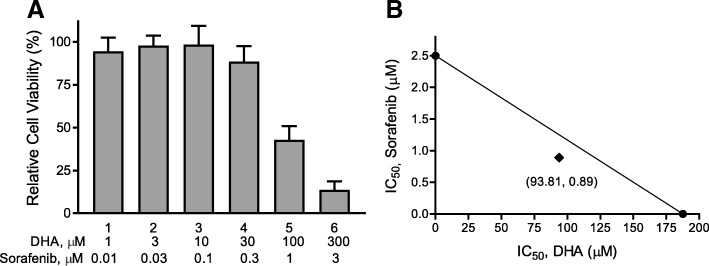
Sorafenib and DHA act in synergy to suppress MDA-MB-231 cell viability. **a** Cells were treated with increasing concentrations of Sorafenib and DHA at a fixed ratio of 1:100, determined by the IC_50_ values of each compound added to cells alone. Cell viability was analyzed using the MTS assay. **b** An isobologram was plotted using the IC_50_ values of Sorafenib and DHA alone on the **Y**- and **X**-axes, respectively. Experimentally derived values from combination treatment lying beneath the addition line indicate a synergistic interaction (*n* = 3)

MDA-MB-231 is a well-established triple negative breast cancer cell line. We have previously shown that this cell line is more resistant to DHA’s cytotoxicity [13]. Currently, there is no effective targeted-therapeutic treatment strategy for triple negative breast cancer as compared to other subtypes of breast cancer, such as hormone receptor positive or Her2/neu breast cancer. We therefore focused on MDA-MB-231 cells to characterize the effects of the combination of Sorafenib and DHA on cell viability. The combination of Sorafenib and DHA enhanced the cytotoxicity upon MDA-MB-231 cells in a concentration- (Fig. 4a) and time-dependent manner (Fig. 4b). The concentration-dependent curve was significantly shifted to the left, suggesting a synergistic interaction of Sorafenib and DHA on MDA-MB-231 cell viability. This was confirmed by an Isobologram analysis showing the synergistic action of Sorafenib and DHA (Fig. 5a-b). The IC_50_ of DHA on suppressing MDA-MB-231 cell viability was reduced from 188 μM to 93.8 μM, and that of Sorafenib from 2.5 μM to 0.89 μM, indicating a synergistic interaction.

### Sorafenib enhances the fish oil-induced suppression of tumor growth in a xenograft nude mouse model

**Fig. 6:**
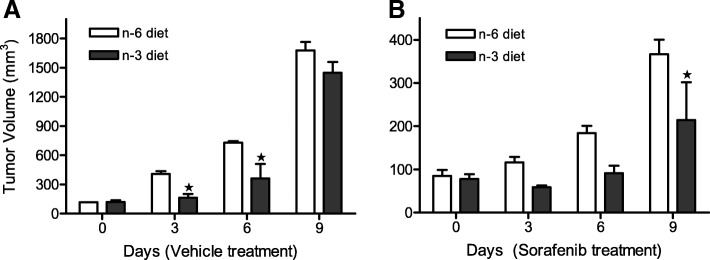
Sorafenib and DHA act together to suppress xenograft tumor growth. MDA-MB-231 cells were implanted into the flanks of nude mice fed either an n-3 or n-6 enriched diet. The xenograft tumor growth, indicated by tumor volume, in mice that were treated either with the vehicle alone (**a**) or with Sorafenib (**b**) are shown (*n* = 5, means ± SEM, *p < 0.01)

To test whether Sorafenib enhances DHAs’ anticancer activity in vivo, we implanted MDA-MB-231 cells into nude mice and examined the effects of Sorafenib on tumor growth in the mice fed either a 7.5% fish oil (high n-3 fatty acids/DHA) or 7.5% corn oil (high n-6 fatty acids), see [23, 32]). As shown in Fig. 6a, compared to corn oil diet the fish oil diet suppressed tumor growth in the xenograft model system, consistent with our recent reports [23, 32]. The addition of Sorafenib significantly enhanced the suppression of tumor growth in the fish oil diet fed mice (Fig. 6b) without affecting mouse weight (data not shown), indicating that the combination of Sorafenib and DHA is a potential new strategy for the treatment of triple negative breast cancer.

### Screening of the oncology drug set IV identifies a set of anticancer compounds, including Sorafenib, that act to enhance DHAs’ cytotoxicity in MDA-MB-231 cells

We have previously reported that DHA acts in concert with clioquinol [30] and disulfiram [32] to more effectively kill cancer cells, suggesting that different mechanisms are involved in the synergistic action of DHA and these anticancer compounds. To further test this assumption, we screened the Oncology Drug Set IV using MDA-MB-231 cells. Interestingly, 48 compounds were found to enhance, while 32 compounds attenuate, and 21 compounds have no effect on DHA-induced cytotoxicity in MDA-MB-231 cells (Table 1). The selected compounds which significantly altered DHA’s cytotoxicity in MDA-MB-231 cells are shown in Table 2. Two tyrosine kinase inhibitors, Sorafenib and Axitinib were among the anticancer compounds that enhanced cytotoxicity when combined with DHA, supporting the idea that tyrosine kinase inhibitors may suppress the cellular antioxidant response potential thereby enhancing DHA’s cytotoxicity. The fact that Imatinib, a more specific tyrosine kinase inhibitor, actually significantly attenuated cytotoxicity when combined with DHA suggest that not all tyrosine kinase inhibitors act the same way in our model system. These observations indicate that further studies are needed to identify various cellular mechanisms that mediate the combined effect of DHA and chemotherapeutic drugs against cancer, in order to develop more effective combination therapies.

**Table 1:** Anticancer drugs have different effects on DHA’s cytotoxicity in cancer cells

MTS assay	Cytotoxicity enhanced	Cytotoxicity attenuated	Cytotoxicity unchanged
Initial screen	48	32	21
Validation^a^	21	20	4

^**a**^Only 45 drugs available for the validation screen

MDA-MB-231 cells were treated with individual drugs (101 anticancer drugs, the NCI Oncology Set IV) at IC_25_ and IC_50_ drug concentrations, and DHA (at 50 μM for initial screen and 25, 50, and 100 μM for validation) for 48 h. Cell viability was determined by MTS assay

**Table 2:** Selected anticancer drugs that alter cytotoxicity when combined with DHA in MDA-MB-231 cells

Enhanced cytotoxicity^a^		Attenuated cytotoxicity^b^	
Drug code	Drug name	Drug code	Drug name
92859	Arsenic trioxide	63878	Cytarabine
747971	Sorafenib	756645	Crizotinib
757441	Axitinib	743414	Imatinib
296961	Amifostine	3053	Dactinomycin
8806	Melphalan	180973	Tamoxifemn
698037	Pemetrexed Disodium	266046	Oxaliplatin
747974	Raloxifene HCl	721517	Zoledronic acid
754143	Romidepsin	719276	Fulvestra

^a^*p* < 0.01, ^b^*p* < 0.05

MDA-MB231 cells were treated with individual drugs at concentrations of IC_25_ and IC_50_ and DHA at 25, 50, and 100 μM for 48 h. Cell viability was determined by MTS assay. Significant enhancement or attenuation of DHA’s cytotoxicity was determined by two-way ANOVA followed by paired analysis

## Discussion

The most interesting finding from the present study was that the small molecule tyrosine kinase inhibitor Sorafenib reverses DHA-induced suppression of nuclear Bach1 expression, thereby attenuating HO-1 induction by DHA. Consequentially, the combination of DHA and Sorafenib led to a synergistic interaction in suppressing cancer cell viability. Since Sorafenib is a well-established anticancer drug [40], and DHA is a dietary compound that possesses great health benefits and is also used clinically [7–9], our findings indicate that the combination of Sorafenib and DHA is an attractive new strategy for more effective cancer therapy.

DHA’s anticancer activity and its mechanisms of action have been extensively investigated over the last several decades [11, 41, 42]. Although the combination of long chain n-3 PUFA, including DHA, and chemotherapeutics for cancer therapy has been described in recent years in various experimental model systems [30, 41, 42] and in clinical trials [11], the potential mechanisms of how DHA might interact with other therapeutics to achieve high therapeutic efficacy remains poorly understood. This lack of understanding limits the further development of combination therapies using DHA and other anticancer drugs. We have previously reported that DHA induces apoptosis of cancer cells primarily through enhanced lipid peroxidation [13], indicating the importance of the cellular antioxidant enzyme system in mediating DHA’s anticancer action. It is well established that cancer cells are more vulnerable to oxidative stress than normal cells. This is thought to be due to a constant increase in cellular levels of reactive oxygen species and an already stressed antioxidant response, thus providing a strategy to selectively kill cancer cells by further enhancing cellular oxidative stress [43–45]. In our recent report, we demonstrated that DHA induces HO-1 gene transcription by promoting Bach1 protein nuclear exportation and degradation. The loss of Bach1 protein allowed an increase in Nrf2 binding to the AREs in the HO-1 gene promoter and activation of HO-1 gene transcription [23]. HO-1 is an antioxidant enzyme that is coupled by the Nrf2 signaling pathway [19], and has been shown to be over-expressed in cancer tissues [22] and contribute to chemo resistance [46, 47]. Thus targeting HO-1 is a potential strategy for cancer therapy [21, 22]. Because Bach1 protein nuclear exportation and subsequent degradation is controlled by tyrosine phosphorylation [26], we assumed that tyrosine kinase inhibitors would reverse DHA-induced Bach1 degradation and block HO-1 induction by DHA, thus leading to enhanced cytotoxicity upon cancer cells. The results from the present study confirmed our hypothesis by testing the combination of the tyrosine kinase inhibitor Sorafenib and DHA in our model systems. Both western blot and reporter gene assay revealed that Sorafenib is able to reverse DHA-induced suppression of nuclear Bach1 expression and induction of HO-1 gene transcription. Furthermore, Sorafenib was found to enhance DHA’s cytotoxicity in various human cancer cell lines and further suppressed xenograft tumor growth in vivo in fish oil fed mice. The combined drug interaction of DHA and Sorafenib was synergistic as evidenced by Isobologram analysis. These findings clearly indicate that targeting Bach1/Nrf2-mediated HO-1 gene expression enhances DHA’s cytotoxicity. This study supports further development of a new combination cancer therapy using DHA and Sorafenib, both being well tolerated in patients and approved by the FDA for clinical applications.

It should be noted that Sorafenib has been described as an inhibitor to the soluble epoxide hydrolase, an enzyme that functions in converting active lipid epoxides to inactive diols [48]. The possibility that Sorafenib inhibition of the soluble epoxide hydrolase results in more active DHA-derived epoxydocosapentaenoic acids thereby enhancing DHA’s cytotoxicity in our model system cannot be excluded. It is indeed plausible that the combination of DHA and Sorafenib could weaken the cellular antioxidant forces (targeting Bach1/Nrf2-mediated HO-1 gene expression) on the one hand and potentially enhance the oxidative potential (inhibiting soluble epoxide hydrolase to elevate DHA-derived epoxydocosapentaenoic acids) on the other hand, thereby leading to synergistic cytotoxicity against cancer cells. The fact that Sorafenib is a multi tyrosine kinase inhibitor [49] suggests that future studies could reveal even more mechanistic insight in the synergistic anticancer action of DHA and Sorafenib.

While DHA has been shown to enhance the anticancer effect of various cancer therapies, including chemotherapy [11] and radiotherapy [50, 51], it remains unclear whether DHA universally enhances the efficacy of all anticancer drugs, or whether it may antagonize the anticancer effect of certain chemotherapeutics. By determining the combined effect of DHA and the 101 anticancer compounds in the Oncology Drug Set IV, the present study for the first time has shown that DHA selectively enhances the cytotoxicity of certain cancer therapeutics, while antagonizing or having little to no effect on the cytotoxicity of other anticancer compounds. These results underline the importance of better understanding the mechanism of action when DHA is used in combination with other cancer therapeutics. In this regard, the present study has raised a critical issue in DHA-based combination therapy: that not every anticancer drug is suitable in combination with DHA for cancer therapy and drugs should be individually evaluated in the appropriate model system if they are intended to be used in combination with DHA for cancer therapy.

## Conclusion

In conclusion, the present study has identified a new strategy for potential combination cancer therapy using DHA and Sorafenib, and has provided preliminary evidence to suggest that DHA may act either in synergy or as an antagonist with other anticancer compounds to affect tumor growth.
